# On the use of computer‐assistance to facilitate systematic mapping

**DOI:** 10.1002/cl2.1129

**Published:** 2020-11-12

**Authors:** Neal R. Haddaway, Max W. Callaghan, Alexandra M. Collins, William F. Lamb, Jan C. Minx, James Thomas, Denny John

**Affiliations:** ^1^ Mercator Research Institute on Climate Change and Global Commons Berlin Germany; ^2^ Stockholm Environment Institute Stockholm Sweden; ^3^ Africa Centre for Evidence University of Johannesburg Johannesburg South Africa; ^4^ Priestly International Centre for Climate University of Leeds Leeds UK; ^5^ Centre for Environmental Policy Imperial College London London UK; ^6^ EPPI‐Centre UCL Social Research Institute, UCL London UK; ^7^ Amrita Institute of Medical Sciences & Research Centre Amrita Vishwa Vidyapeetham Kochi Kerala India

**Keywords:** automation, evidence map, evidence synthesis, evidence synthesis technology, machine learning

## Abstract

The volume of published academic research is growing rapidly and this new era of “big literature” poses new challenges to evidence synthesis, pushing traditional, manual methods of evidence synthesis to their limits. New technology developments, including machine learning, are likely to provide solutions to the problem of information overload and allow scaling of systematic maps to large and even vast literatures. In this paper, we outline how systematic maps lend themselves well to automation and computer‐assistance. We believe that it is a major priority to consolidate efforts to develop and validate efficient, rigorous and robust applications of these novel technologies, ensuring the challenges of big literature do not prevent the future production of systematic maps.

## 1 INTRODUCTION: THE NEED FOR TECHNOLOGY IN EVIDENCE SYNTHESIS

**Figure 1:**
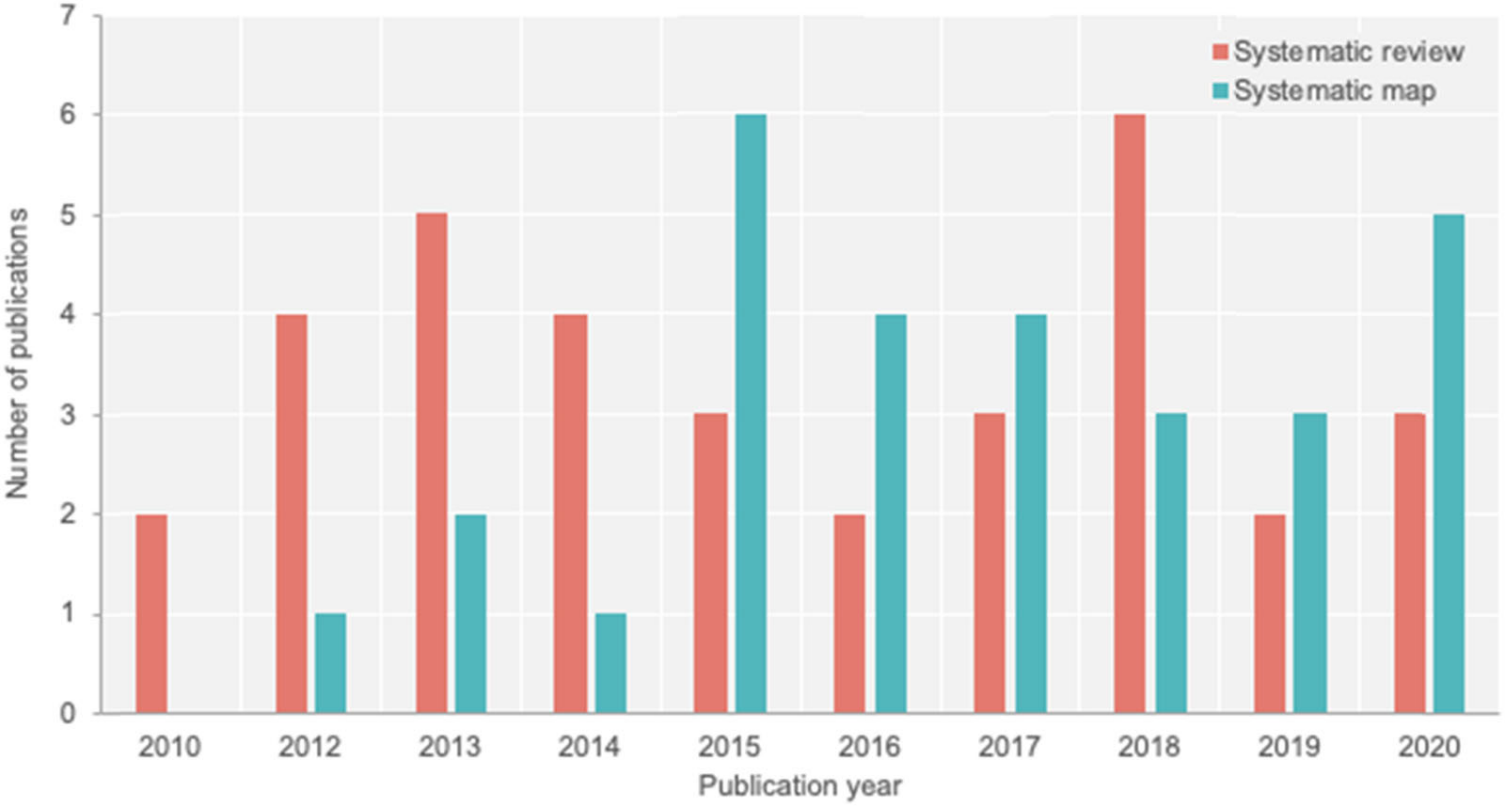
Comparative growth in systematic maps versus systematic reviews over time in the journal *Environmental Evidence* (as of 10.06.2020)

The volume of published academic research is growing rapidly, and in some topics exponentially (Bornmann & Mutz, 2015; Khabsa & Giles, 2014). This new era of “big literature” (Callaghan et al., 2020; Minx et al., 2017; Nunez‐Mir et al., 2016) poses new challenges to evidence synthesis, pushing traditional, manual methods of evidence synthesis (systematic reviews and systematic maps; Gough et al., 2017) to their limits. At the same time, evidence mapping approaches (systematic maps (James et al., 2016), evidence and gap maps (Snilstveit et al., 2013), evidence maps (Saran & White, 2018); see Box 1) are becoming more popular than full syntheses in some disciplines, such as in environmental science (Figure 1). These approaches that aim to catalogue broad evidence bases typically deal with greater volumes of evidence than traditional systematic reviews and are thus even more sensitive to increasing publication rates. This increase in breadth and procedural reduction means that mapping is inherently more scalable, however. To illustrate, the average number of search results in recent environmental systematic maps is more than 34,000 (Haddaway & Westgate, 2019). A typical systematic map also requires in excess of 200 person‐days to complete—a significant time and resource investment (Haddaway & Westgate, 2019).

Box 1 **What is systematic mapping?**

*Overview*: Systematic mapping is an evidence synthesis method similar to systematic reviewing that aims to summarise an evidence base using robust and rigorous processes. However, systematic mapping differs from systematic reviewing in that it does not aim to summarise the findings of the studies in the evidence base, but rather summarise the evidence base as a whole. While systematic reviews typically focus on answering questions like “what works, when and for whom”, systematic maps aim to answer “what do we know about a topic”, or “what research exists on a particular area”. Systematic maps are often conducted as a first step in the *evidence synthesis pathway*, and would be followed by a number of focused systematic reviews that could be conducted relatively swiftly following the mapping work already undertaken (searching for, screening and describing studies).

*Methods*: Systematic maps follow the same procedures as systematic reviews, but differ at the stage of extraction of information until the synthesis of studies. Systematic maps typically extract only meta‐data (descriptive information about the studies and their systems) and apply predefined codes: they do not extract findings from the included studies. Systematic maps may include critical appraisal of included study validity, but this step is optional and would be conducted in full in subsequent systematic reviews. Synthesis in systematic maps involves a description of the evidence base and development of an interactive database of studies, and therefore does not include the quantitative or qualitative synthesis of study findings.

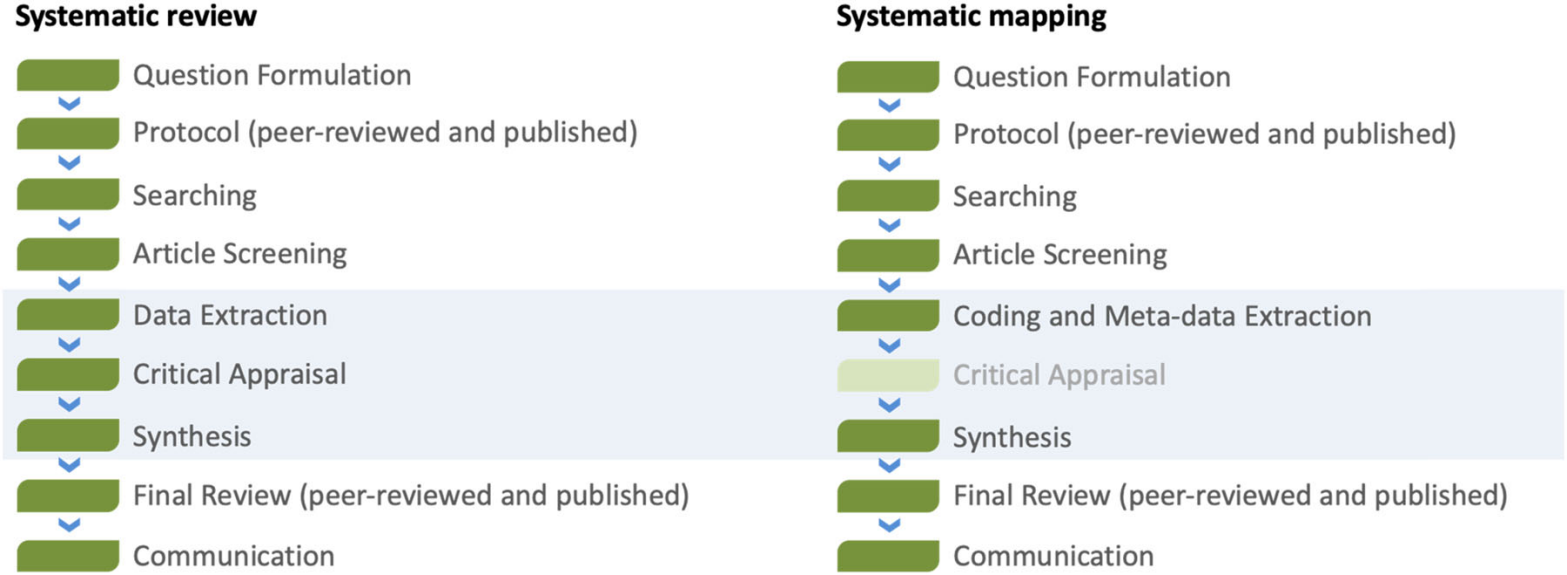

*Outputs*: The key outputs of a systematic map are: (a) an interactive, searchable database of relevant studies and their descriptive information; (b) a list of knowledge gaps (topics where primary research is limited or absent) and knowledge clusters (topics where sufficient research exists to warrant full synthesis via systematic reviews); (c) visualisations of the evidence base to facilitate understanding (e.g., evidence atlases, heat maps, descriptive plots); (d) a summary report describing the background, methods and results.

*Terminology*: The term “systematic map” is widely used in environmental science and social science. Some fields refer to this method as “evidence maps”, although this is perhaps a broader definition, procedurally. In the field of social welfare, the Campbell Collaboration has developed the term “evidence and gap map” to describe a form of evidence map that focuses on identifying systematic reviews (and often impact evaluations) on a topic, and developing a matrix of interventions and outcomes to visually represent the evidence base. In the field of health, an analogous approach would be described as a “scoping review”, but this term includes a broader set of methods that include reviews conducted to a lower standard than systematic maps (Colquhoun et al., 2014). Furthermore, the term “scoping review” is used to describe a preliminary, exploratory step prior to the conduct of a full evidence synthesis in many fields (CEE, 2013).

*Example*: In their systematic map of the effects of nature conservation on human wellbeing, McKinnon et al. (2016) retrieved 35,782 search results, resulting in 1,043 relevant records being retained in their final systematic map. The authors produced a series of visualisations including heat maps and an evidence atlas (an interactive geographical map of the evidence base) that described the spread of evidence across regions, conservation actions and measured outcomes (among other variables). They also included a searchable systematic map database describing all included studies as a supplementary file. An interactive, online version of their systematic map can be explored here: https://www.natureandpeopleevidence.org/#/explore/wellbeing/charts.

Further guidance and analysis of the uses and benefits of systematic mapping can be found here:

James, K. L., Randall, N. P. & Haddaway, N. R. A methodology for systematic mapping in environmental sciences. *Environmental Evidence* **5**, 7 (2016). https://doi.org/10.1186/s13750-016-0059-6

Haddaway, N. R., Bernes, C., Jonsson, B. et al. The benefits of systematic mapping to evidence‐based environmental management. *Ambio* **45**, 613–620 (2016). https://doi.org/10.1007/s13280-016-0773-x

Wolffe, T. A. M., Vidler, J., Halsall, C., Hunt, N., Whaley, P., A survey of systematic evidence mapping practice and the case for knowledge graphs in environmental health and toxicology, *Toxicological Sciences* **175**, 35–49 (2020). https://doi.org/10.1093/toxsci/kfaa025

McKinnon, M. C., Cheng, S. H., Garside, R., Masuda, Y. J., Miller, D. C., Sustainability: Map the evidence. *Nature News*, **528**, 185 (2015). https://doi.org/10.1038/528185a

Saran, A., White, H., Evidence and gap maps: a comparison of different approaches. *Campbell Systematic Reviews*, **14**, 1–38 (2018). https://doi.org/10.4073/cmdp.2018.2

We focus here on systematic mapping for two reasons (see Box 1 for a definition). First, mapping approaches are ubiquitous across evidence synthesis methodology, despite not formally being recognised: many systematic reviews also involve an initial mapping stage that identifies, collates and describes the nature of the evidence base. Second, systematic mapping methods focus on the location and description of corpora of literature: methods that lend themselves well to computer assistance and automation. The latter stages of systematic reviews (including data extraction, critical appraisal and synthesis) require in‐depth analysis of individual manuscripts that is so far proving highly challenging for automation. The approaches described herein could thus be relevant to a range of different evidence synthesis and mapping methods.

Despite this growing problem, there are signs that new technology developments, including machine learning, are likely to provide solutions to the problem of information overload (Zarocostas, 2020) and allow scaling of evidence syntheses to large and even vast literatures (Callaghan et al., 2020; Lamb et al., 2019; Westgate et al., 2018). First, such technologies may enable reviewers to deal with vast evidence bases far more efficiently and swiftly than humans alone, by bearing the burden of repetitive and routine tasks and allowing manual efforts to be diverted towards more complex tasks. Second, they can identify patterns and content across large sets of documents that would otherwise be inaccessible or incomprehensible to humans, breaking new ground in evidence synthesis and knowledge aggregation. Thirdly, they offer new opportunities for identifying additional relevant literature within given resource constraints, while also potentially enhancing the consistency and accuracy of screening, study selection and coding. Finally, they may help to accelerate the process of conducting systematic maps. Computer‐assisted reviews thus offer the promise of increased efficiency, scientific insight, comprehensiveness, rigour and speed.

In this paper, we outline how systematic mapping methods lend themselves well to automation and computer‐assistance, and highlight evidence synthesis technology as a vital means of dealing with the rapid expansion of evidence across disciplines (so‐called *infodemics* (Zarocostas, 2020). We argue that a major priority is to consolidate efforts to develop and validate efficient, rigorous and robust applications of these novel technologies. In doing so, we will ensure the challenges of big literature do not prevent the future production of systematic maps.

## 2 THE BENEFITS OF SYSTEMATIC MAPPING

Systematic mapping aims to catalogue and describe the evidence base, unlike systematic reviews, which aim to extract and synthesise study findings. Systematic maps are typically used to provide an overview of a broader landscape of research, and as such are useful as a first step on the “evidence synthesis pathway”: the process of moving from broad stakeholder concerns (“what do we know about…”) to a series of focused answers to targeted questions about impacts and “what works” (Gough et al., 2017). Often, the scope of systematic maps more closely aligns to the broad concerns fielded by commissioners and other stakeholders: certain aspects of the key elements of systematic maps (populations, interventions/exposures, comparators, outcomes, methods/study types) are typically included iteratively and are not restricted by prespecified inclusion criteria. Because of this and by design, systematic mapping can be used to catalogue multiple types of evidence in the same map: modelling studies, laboratory studies, field studies, quantitative research, qualitative research, commentaries, theories and methodologies.

Although they do not synthesise study findings, systematic maps are an important means of highlighting knowledge gaps (subtopics where primary research is lacking), knowledge clusters (subtopics where sufficient studies exist to allow full synthesis via systematic review) and best‐practices and/or deficiencies in research methods. They can thus help to direct impactful and needs‐driven research and funding towards primary and secondary gaps in the evidence.

Systematic maps can be upgraded into full systematic reviews relatively quickly for given knowledge clusters and, in these cases, can be particularly resource efficient. They can thus be used to determine for which subtopics systematic reviews are most appropriate and feasible, reducing the risks of undesirable empty reviews.

**Figure 2:**
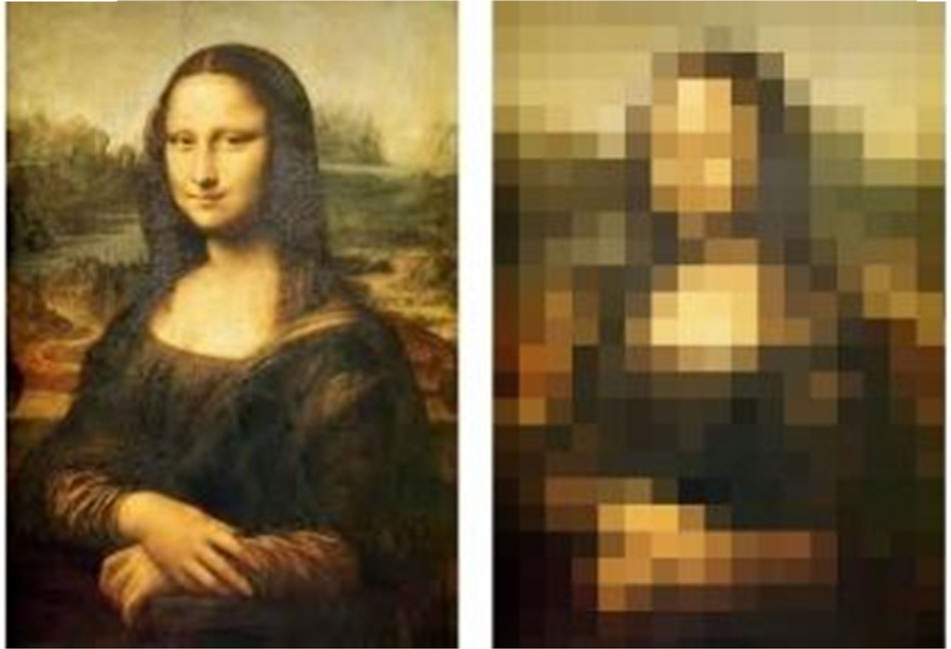
The Mona Lisa and a pixelated version, still easily recognisable. Image Source: https://www.newmediacampaigns.com/page/how-to-optimize-an-image-for-your-website

Representativeness is arguably more important than comprehensiveness in systematic maps, since the aim is to identify patterns in gaps and clusters rather than generate precise estimates: as an analogy, a photograph of the Mona Lisa is recognisable at low resolution, but it could not be considered an accurate representation of the underlying information in the painting (Figure 2). This reduces the risks of non‐exhaustive searching in systematic maps: in systematic reviews, there can be severe consequences if even one relevant study is missed, but this is not the case for maps, which summarise broad patterns. Furthermore, there are minimal risks of including a small number of irrelevant studies in a map, since it is broad patterns that are of interest, and further screening and critical appraisal is necessary before proceeding to full synthesis in any subsequent systematic review. This relaxation of the exacting standards traditionally required of systematic reviews makes computer‐assisted (and perhaps even automated) maps more viable.

## 3 **HOW** *EVIDENCE SYNTHESIS TECHNOLOGY* CAN SUPPORT SYSTEMATIC MAPPING

There are many key points in the traditional view of systematic mapping processes where evidence synthesis technology and computational methods could be applied, broadly speaking, project management, retrieval, screening, data extraction and narrative synthesis. We discuss these processes in the following sections.

### 3.1 The benefits of computer‐assisted mapping to research, policy and practice

The computer‐assisted (or automated) systematic mapping that we describe herein has a suite of benefits to researchers, policymakers and practitioners. More rapid mapping of the evidence will result in faster synthesis in general (including the transition from systematic maps to systematic reviews), by facilitating the identification and analysis of the nature of an evidence base. In turn, this will allow evidence synthesis to be more responsive to the knowledge needs of decision‐makers. Where decision‐makers require urgent evidence on a subject, computer‐assisted mapping can make the provision of a timely *and* systematic map, where previously evidence synthesis would have had to be either timely *or* systematic. In addition, because computer‐assisted systematic maps can be continually updated to include relevant research as and when it is made available, maps can be kept up to date with the latest evidence.

### 3.2 Current technologies

We highlight below some key examples of *evidence synthesis technologies* (defined here as novel tools and frameworks used to support evidence syntheses) that currently exist and can already be used to increase the efficiency, rigour and accessibility of evidence synthesis. We explain these across the main processes of systematic mapping.

1. **Project management**—In its broadest form, review management tools are evidence synthesis technologies that suit themselves well to systematic maps, since they support an entire flow of information through the review process, can deal with *big data* review projects, and facilitate good record keeping, which is vital with such large evidence bases.
2. **Retrieval**—Text analysis processes can suggest search terms to support search string development based on computer‐assisted scoping of the literature (Grames et al., 2019; Stansfield et al., 2017). Text analysis processes can be used to retrospectively improve on Boolean search strings to improve search strategies for updates to bibliographic searching. Similarly, text analysis and citation network processes (e.g., Weller et al., 2018) can be used to suggest additional relevant articles that have been published since searches were conducted to update systematic review searches, ensuring the longevity of the map, which would otherwise become out‐of‐date as soon as searches are concluded.
3. **Screening**—Machine learning tools can help to increase efficiency in screening by prioritising relevant research so that eligible studies can be found early on in the screening process and other stages (meta‐data extraction) can begin sooner (O'Mara‐Eves et al., 2015), although traditionally, all records should be screened by at least one human (CEE, 2018). Recent methodological developments have suggested processes of transparently cutting off screening processes at given levels of confidence about the completeness of the process (Callaghan & Müller‐Hansen, 2019). In addition, named entity recognition can be used to identify terms from a predefined dictionary or taxonomy to support screening: for example, Lamb et al. (2019) used a dictionary of city names to identify case studies from the climate literature.
4. **Data extraction**—Topic modelling and machine learning classifiers can be used to interrogate the body of evidence in order to gain a relatively crude understanding of the content and focus of relevant research (Jaspers et al., 2018; Stansfield et al., 2013; Westgate, 2019). Unsupervised machine learning can generate document labels without human input, which can be interpreted by humans and mapped to other document metadata (Lamb et al., 2019). Supervised learning can be used to rapidly label large collections of documents based on labels given by humans to a subset of documents (Mcauliffe & Blei, 2008).
5. **(Narrative) synthesis**—Visualisation of systematic map databases using cartographic/conceptual mapping tools can be used to understand the content and spread of studies of geographical/conceptual space (e.g., Lamb et al., 2019; van Soest et al., 2019).

### 3.3 How technology could redefine evidence synthesis methods

**Figure 3:**
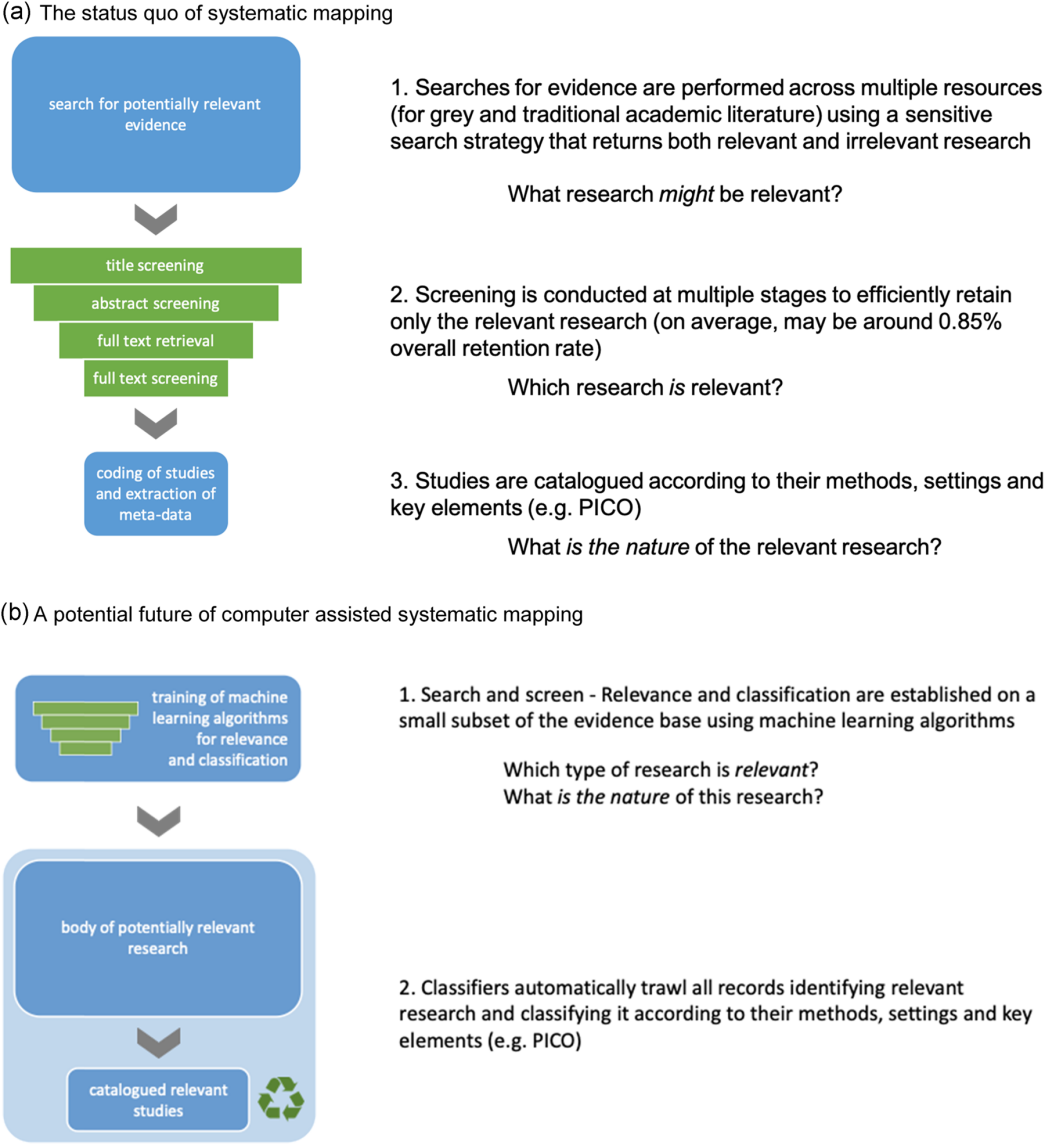
Current and future approaches to document discovery, eligibility screening and cataloguing. (a) The status quo of systematic mapping. (b) A potential future of computer‐assisted systematic mapping

Evidence synthesis methods at present separate the processes of searching and screening to maximise first sensitivity and then specificity, somewhat independently: they gather a large body of potentially relevant evidence by searching for specific terms that must be mentioned, and then narrow this corpus down by manually assessing and extracting explicit and implicit meanings (Figure 3a).

This approach is necessary, but time consuming and inefficient. It is necessary because, at present, sensitive searches are the only way to ensure all available records are retrieved. It is inefficient because a large volume of information must be read and screened for possible relevant, with the vast majority being discarded (only 8% [*n* = 11] of relevant, deduplicated search results were included after title and abstract screening, and only 3% [*n* = 17] after full‐text screening in a recent assessment of systematic reviews and maps (Haddaway & Westgate, 2019).

Evidence synthesis technology could allow us to blend the processes of searching, screening and coding into a single, organic research discovery step by developing algorithms to predict document relevance based on trialled and validated human assessments of relevance, blended with other methods like citation/collaboration network analyses (Figure 3b).

Developing a blended research discovery process in this way, could help to develop curated bodies of evidence where the information generated through the act of reviewing is shared across researchers and project, instead of wasting effort across reviews and having to repeat the work of screening vast numbers of potentially relevant records. The use of automation in cataloguing this evidence makes this more feasible by drastically reducing the efforts needed to screen and classify studies. This iterative process of blended searching and screening, could drastically increase comprehensiveness and efficiency, and reduce research waste.

In healthcare, technology is being harnessed to automate several steps in the evidence synthesis process, collating, summarising and appraising all randomised control trials (TrialStreamer; https://trialstreamer.robotreviewer.net/).

### 3.4 Facilitators to automated systematic mapping

We describe here what we believe to be some of the key areas that require development to support further computer‐assistance and full automation of systematic mapping.

1. **Open Discovery**—Large, open (i.e., free‐to‐access) bibliographic datasets could be developed and curated with supplementary information related to their contents and relation to the rest of the evidence base, for example, across broad conceptual topics such as climate change. Such open datasets would facilitate digestibility by automated processes and sharing of information across reviews that would otherwise be unnecessarily replicated. Bulk downloading should be a priority to reduce reviewer workloads and potential errors. For example, Lens.org is a free‐to‐use bibliographic database that aggregates across Microsoft Academic, CORE, CrossRef and PubMed, cataloguing >220 m academic records. It allows bulk exporting of up to 50,000 records in one go.
2. **Machine readable, accessible full texts**—Text analysis techniques (including machine learning) are mostly based on abstracts, but developing comprehensive corpora of full texts or key portions of full texts (the most likely areas of relevance are objectives, methods, results) would, in some cases, greatly facilitate more comprehensive and robust technology‐assisted discovery of relevant evidence and analysis of article content.
3. **Standardised classification frameworks and ontologies**—Text analysis methods are more powerful and have broader applicability when combined with established ontologies or classification frameworks for concepts and their synonyms (e.g., how different interventions for treatment of lung pathologies are interrelated and described).
4. **Data extraction/coding**—Automated extraction of meta‐data from full texts is a key challenge but would speed up a time‐consuming process and should focus on all aspects of the map's key elements, including study location, population type, intervention type and strength, comparator type, measured outcome, context, study design and methods. Some of this information is easily identified through text analysis (e.g., using dictionary methods; Welbers et al., 2017), but some may be more challenging to identify and extract, for example, the context, study design and methods.
5. **Scientometric mapping**—The integration of scholarly networks (who works with whom, who cites whom) and systematic mapping to facilitate computer‐assisted “research weaving” (analysis of the who as well as the what; Nakagawa et al., 2018) will allow for more efficient and comprehensive discovery of relevant research.
6. **Off‐the‐shelf classifiers**—The establishment of public machine learning libraries (classifiers) of broad concepts (e.g., “developing nations”) for use in screening and coding that can be validated will facilitate rapid and comprehensive discovery and analysis of relevant evidence (e.g., Marshall et al., 2018).
7. **Integration and validation**—The development of linkages with crowd‐sourcing initiatives (e.g., Cochrane Crowd; https://crowd.cochrane.org) would facilitate large‐scale validation of automation technologies, by allowing methodologists to compare the results of computer‐driven procedures with human‐driven results.
8. **More collaborative development**—To harness the increasing global attention focused on the use of machine learning and other technologies in text analysis, *evidence synthesis technologists* (those developing tools and frameworks for automation and computer‐assistance) should work collaboratively to share data and methodological protocols and avoid wasted effort. Organisations like the Evidence Synthesis Hackathon (www.eshackathon.org) and the International Collaboration for the Automation of Systematic Reviews (icasr.github.io) are paving the way.
9. **Development of different types of maps**—If routine tasks can be highly automated and large literatures are at least crudely machine‐synthesisable, systematic maps may no longer be limited to narrow research questions. This opens the door to very ambitious mapping efforts, at the scale of disciplines or major topic areas (Callaghan et al., 2020).

## 4 BARRIERS AND FACILITATORS TO COMPUTER‐ASSISTED SYSTEMATIC MAPPING

Such transformations to the way that we conduct evidence syntheses are not without their challenges. We highlight some of the major barriers below and suggest factors that may facilitate the transition to more computer‐assisted systematic mapping.

### 4.1 Technical issues

1. **Lack of interoperability**—At present, tools are rarely interoperable, although there is some standardisation of citation formats. Automated systematic mapping requires that existing and future technologies are interoperable (i.e., use compatible file formats and data standards) to facilitate multiteam tool development and collaboration (and avoid redundancy and development waste).
2. **Paywalls**—Many effective tools with long‐term support must charge subscription or access fees to ensure their longevity. These paywalls may be restrictive for reviewers working in resource‐constrained contexts. Waivers for low‐ and middle‐income country users and institutional subscriptions can help to support wider use, however.
3. **Lack of open discovery**—Automation of systematic mapping requires comprehensive access to large bodies of abstracts and full texts. However, this big data is typically held by publishers and in bibliographic databases that are hidden behind paywalls, or have built‐in use restrictions that limit the volume and type of data that can be accessed. Some novel platforms and databases, such as Microsoft Academic (https://academic.microsoft.com/home), Crossref (https://www.crossref.org/), and Lens.org (https://www.lens.org/), do allow large‐scale access to bibliographic information (so‐called Open Discovery). Open Access (freely accessible full texts) is increasing but is not yet sufficient for use in automated mapping. Even when researchers do have access to papers, obtaining full texts at scale and in a machine‐readable format is rarely possible.
4. **Lack of standardised validation**—Many tools require robust and independent validation before they are regarded as being safe to use. However, validation has not yet been standardised and is typically self‐assessed by those producing evidence synthesis technologies. In order to validate tools, there is a need for “gold standard” datasets from example reviews, which have been scarce to date (O'Connor et al., 2019).
5. **Challenge in extracting implied meaning**—Coding and extraction of meta‐data from full texts require a detailed understanding of nuance (i.e., interpretation of implied meaning rather than explicit terminology) that is not yet fully feasible with automation technologies because of the wealth of information in research articles and the nuance and implicit meanings hidden within narrative style documents. For example, the presence of a phrase in an article's methods text does not necessarily mean that method was used; “We chose to use a blocked design because of the challenges associated with randomised control trials”.

### 4.2 Cultural barriers

1. **Coding skill barrier**—Currently, tool use sometimes requires knowledge of coding or programming ability, for example, many of the packages in the R environment (e.g., litsearchr, Grames et al., 2019). Graphical user interfaces (GUIs) allow users to interact with tools without requiring programming skills, but typically require substantial resources to create. Tools like Shiny (https://www.shinyapps.io/) allow standardised GUIs to be produced for R packages with relative ease, reducing necessary resource investment while increasing usability.
2. **Perception/trust barrier**—In general, people do not yet trust machines to replace humans in research tasks (O'Connor et al., 2019): this may be potential reviewers, editors, peer reviews or readers. However, it should be clear that computer‐assisted systematic mapping can provide a range of tools to select from and support human reviewers, including tools to conduct laborious, routine tasks. This then provides more time for the interpretative and human‐driven elements of synthesis.
3. **“Handing over to computers” barrier**—If humans are used for an initial mapping and technology keeps processes up‐to‐date, what standards should be in place to ensure consistency and accuracy over time? At what point do we hand over to technology, and what procedures should be in place for verifying that they are still functioning appropriately? These questions must be answered before broad‐scale automated systematic mapping can be rolled out.

## 5 CONCLUSIONS

A wide variety of tools already exist to support computer‐driven systematic mapping. These tools sit on a spectrum from those intended to facilitate human‐driven mapping, to those intended to replace humans entirely. However, current technology is not yet sufficient to automate all of the processes involved in rigorous evidence synthesis. Furthermore, rigorous validation is necessary before many of these tools can be trusted for wide‐scale use by the broad research community.

We have highlighted some of the main opportunities that already exist, where the key intervention points are in systematic mapping, and what developments in technologies and frameworks are needed to facilitate the increased use of evidence synthesis technology in systematic mapping. We believe that systematic mapping lends itself well to at least partial automation and call for the methodology and tool development community to collaborate to reduce redundancy and increase the efficiency of evidence synthesis technology development and integration.
